# Physiologically-based pharmacokinetic model to predict loading dose polymyxin B exposure in critically ill patients with sepsis

**DOI:** 10.3389/fphar.2026.1827712

**Published:** 2026-09-01

**Authors:** Yixuan Cao, Inna Galvidis, Akmal Alimov, Joseph F. Standing, Maksim Burkin, Yury Surovoy

**Affiliations:** 1 University College London, London, United Kingdom; 2 I. Mechnikov Research Institute for Vaccines and Sera, Moscow, Russia; 3 Federal Center for Treatment and Rehabilitation Ministry of Health, Moscow, Russia; 4 Middlesex University, London, United Kingdom; 5 University College London Hospital, London, United Kingdom

**Keywords:** critical illness, loading dose, PBPK, pharmacokinetics, polymyxin B, sepsis

## Abstract

**Objectives:**

To build a physiologically based pharmacokinetic (PBPK) model to predict polymyxin B (PMB) exposure in critically ill patients with sepsis.

**Patients and methods:**

A new PBPK model was built using published data on PMB pharmacokinetics (PK) in healthy volunteers and patients with end-stage renal disease (ESRD). The model was then tested to predict individual PK curves on clinical samples from critically ill patients with sepsis (N = 15) accounting for common pathophysiological alterations observed in this cohort (changes of unbound drug fraction, protein levels, fluids shifts, renal function). The developed model was then used to predict PMB plasma and lung concentrations for probability of target attainment (PTA) analysis.

**Results:**

The PBPK model demonstrated good prediction of mean population PK parameters, including area under the concentration time curve (AUC_0-last_), maximal concentration (C_max_) and clearance (CL), with predicted-to-observed ratios ranging from 0.98–1.2. Mean absolute prediction error (MAPE) and root mean squared error (RMSE) for individual predictions reached 26.7% and 7.7%, although the correlation was moderate (r = 0.47). Based on the predicted plasma exposures only the loading dose of 2.5 mg/kg and above achieved the PTA >90% with MIC of 1 mg/L.

**Conclusion:**

The first PBPK model of PMB in critically ill patients allows good prediction of population-level PK parameters and supports a loading dose of at least 2.5 mg/kg. It also provides a mechanistic framework for future research as PMB distribution, metabolism and excretion mechanisms become better characterised.

## 1 Introduction

Antimicrobial resistance is becoming an increasingly concerning issue for global healthcare and is expected to become the leading cause of mortality by the year 2050 (Tackling drug). This problem becomes especially difficult in critical illness, which makes patients more vulnerable to the infection and impose unique pharmacokinetic variability, making optimal dose recommendations challenging (Roberts et al., 2014a).

Polymyxin B (PMB) is among a few antibiotics which have some activity against carbapenem-resistant strains of *Enterobacteriaceae* and *Acinetobacter baumannii* (Velkov et al., 2013; Zha et al., 2020). However, it is characterized by a relatively narrow therapeutic window and high incidence of toxicity. Polymyxins have been in clinical use since the 1950s and have not undergone rigorous pre-clinical and early clinical studies in line with current regulatory requirements (Velkov et al., 2019), and because of its niche application mostly in critically ill patients the data on its pharmacokinetics (PK) remains sparse.

Critical illness is associated with profound physiological derangement related to sepsis, inflammation and organ dysfunction. This can have dramatic implications on drug exposure, putting the patient at risk of therapeutic failure and toxicity when applying standard dosing regimens. This issue is commonly addressed with therapeutic drug monitoring and pharmacokinetic modelling in specific subgroups of intensive care patients (such as sepsis, renal dysfunction and renal-replacements therapy). Unfortunately, population PK analysis in critically ill is extremely challenging due to small sample sizes, simultaneous presence of multiple PK-altering factors issues and their rapid evolution over time.

In light of these limitations, there is growing interest in physiologically based pharmacokinetic (PBPK) modelling, which provides a framework for integrating prior knowledge such as physicochemical properties of the drug and patient physiology to predict drug concentration both in plasma and other major organ compartments. While initially used in early drug development (Jones and Rowland-Yeo, 2013), PBPK is now finding its role in critical illness research. PBPK has been successfully applied to predict drug exposure in a previously uncharacterized setting, such as fluconazole in pediatric patients receiving extracorporeal membrane oxygenation (Watt et al., 2018), in markedly deranged physiology (vancomycin (Radke et al., 2017) and meropenem (Yang et al., 2024)), and to predict drug penetration and bacterial response in the site of interest (colistin and rifampicin (Zhang et al., 2023), ciprofloxacin (Sadiq et al., 2017)).

The aim of the current study was to develop a new PBPK model to inform the loading dose of PMB in critically ill patients, calibrating the model using available PK data from healthy volunteers and patients with end-stage renal disease (ESRD) (Fang et al., 2024; Liu et al., 2021), as well as mechanistic insights from the animal models (Abdelraouf et al., 2012; Manchandani et al., 2016a). The model was then evaluated using prospective individual level data from critically ill patients and used for prediction of PMB exposure in plasma and lung with probability of target attainment (PTA) analysis.

## 2 Methods

### 2.1 PBPK

**FIGURE 1:**
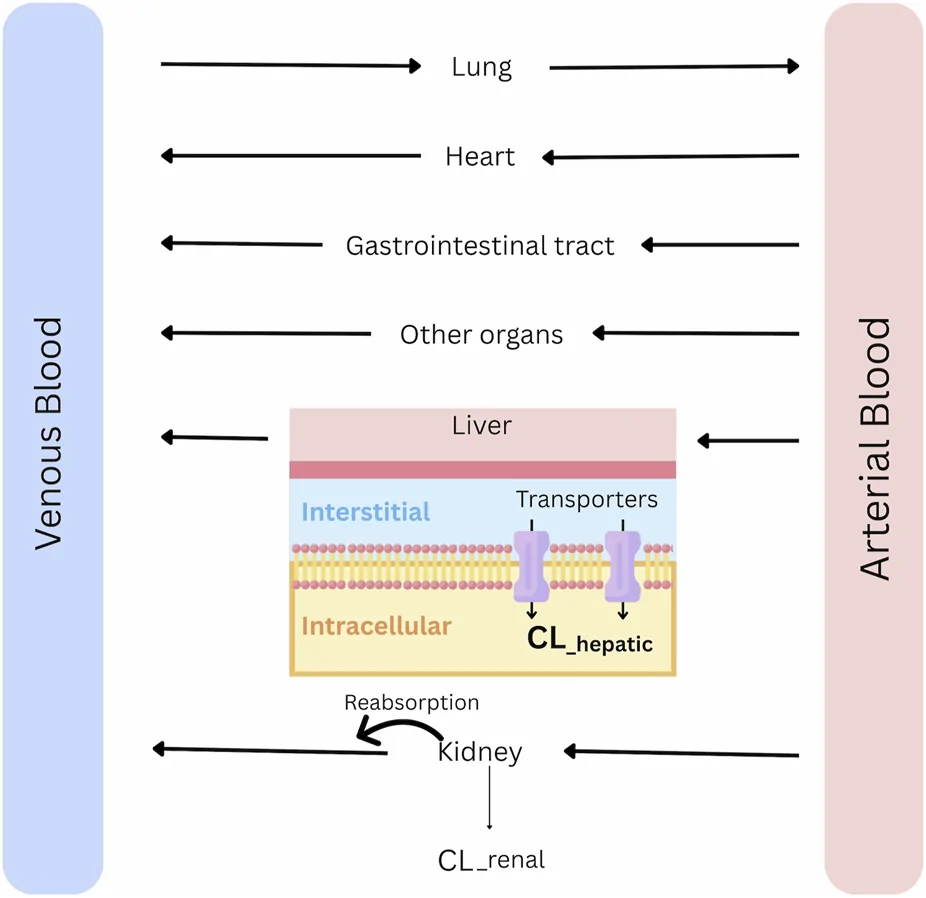
Schematic of the polymyxin B full PBPK model in humans. Arterial and venous blood connect major organs, including lung, heart, gastrointestinal tract, liver, kidney, and other organs. Hepatic drug disposition is represented by transporter-mediated uptake into hepatocytes, leading to hepatic clearance (CL_hepatic). Renal elimination is described by kidney clearance (CL_renal), with tubular reabsorption indicated. Please note, that hepatic transporters represent model assumption rather then true experimental data.

PBPK model development was conducted within PK-Sim 12.1 (Open Systems Pharmacology, United States of America), applying the platform’s dedicated small-molecule workflow. A whole-body PBPK structure for PMB was established as the foundational modelling framework, with the overall architecture shown in Figure 1. Two key processes relevant to the disposition of PMB are represented in the model: hepatic uptake mediated by transporters and renal tubular reabsorption, together resulting in predominantly non-renal elimination of the drug. Model parameterization followed the conventional PBPK classification scheme, comprising drug-dependent physicochemical properties, system-dependent physiological inputs, and population-dependent demographic descriptors. Physicochemical properties of PMB were assumed to remain constant across all simulations (Table 1). Of note, PMB is produced as a mixture of several structurally similar components. In the present study PMB was approached as a single entity using the properties of the principal component B1. Healthy volunteer data was taken from Liu et al., (2021) (Liu et al., 2021) and Fang et al., (2024) (Fang et al., 2024) with mean PMB plasma concentration retrieved through digitisation with WebPlotDigitizer 4 (Automeris LLC, United States of America), population characteristics from these studies are presented in the Supplement (Supplementary Table S1).

**TABLE 1:** Main inputs for PBPK model.

Parameters^a^	Value	Ref
Molecular weight (g/mol)	1,203.50	(DrugBank) (Knox et al., 2024)
log P	−0.89	(DrugBank)
pKa acid	11.6	(DrugBank)
pKa base	10.2	(DrugBank)
Water solubility (mg/ml)	0.0744	(DrugBank)
Protein Ka	15.6	Parameter identification^b^
*fu*	6% Healthy 6% ESRD 35% Critically ill	(Fang et al., 2024) (Fang et al., 2024) (Surovoy et al., 2022)
Partition coefficients	Rodgers and Rowland	(Rodgers et al., 2005)
Cellular permeability	Charged-dependant Schmitt normalized to PK-Sim	(Willmann et al., 2003)
Albumin, g/L	45 Healthy 38 ESRD 17–43 Critically ill	(Willmann et al., 2003) (Heimbac et al., 2021)
Interstitial fluid fraction (fat and muscle)	0.16 Healthy 0.3 ESRD 0.28 Critically ill	PK-Sim standard Parameter identification^b^ (Radke et al., 2017)
Haematocrit	0.47 Healthy 0.37 ESRD 0.37 Critically ill	(Davies and Morris, 1993) (Heimbac et al., 2021) (Radke et al., 2017)
Transporter expression, ratio of healthy	1 Healthy 0.5 ESRD 1 Critically ill	Parameter identification^b^

^a^ The physicochemical properties used for PMB, correspond to the principal component B1. The behaviour of minor components is assumed to be similar.

^b^ Parameter identification in PK-Sim utilizes the Levenberg-Marquardt nonlinear least-squares algorithm (Willmann et al., 2003; Gavin, 2013) to fit prediction to observed data.

ESRD, end-stage renal disease. *Fu*–unbound fraction. Ka–association constant.

### 2.2 Changes of physiological parameters in disease

The initial PBPK model was subsequently adapted to represent patients with ESRD through the incorporation of disease-specific physiological modifications, as presented in Table 1. Mean PMB concentration curve and clinical data for patients with ESRD were retrieved from Fang et al. (Fang et al., 2024). GFR was reduced to 15 mL/min, plasma albumin levels and haematocrit were reduced to approximately 85% and 77% of healthy reference values, respectively (Heimbac et al., 2021). To further describe observed PK data in ESRD the following plausible pathophysiological alterations were introduced: decreased blood and intracellular pH, increased interstitial fluid fraction to represent tissue edema. Transporter expression in the model was reduced to match the decreased clearance observed in patients with ESRD.

For critically ill patients we created 15 individual simulations based on the available clinical and demographic data. For critically ill patients, the PBPK model was developed using population clinical data summarized in Table 2. Some parameters were assumed based on the available data, in that case fixed values were used for every patient. The unbound fraction was fixed at 35% based on data previously reported by our group (Surovoy et al., 2022), with 10% of inter-individual variability, hematocrit at 0.37, fat and muscle interstitial fluid fraction was increased to 0.28 to indicate changes of fluid composition commonly observed in sepsis (Radke et al., 2017). Sensitivity analyses were performed over plausible ranges of these parameters to assess their effects on AUC, Cmax and CL.

**TABLE 2:** Clinical and demographic data.

Parameters	Polymyxin B group
N	15
Age, years	68 ± 4.1 (37–89)
Sex Male Female	11/15 4/15
Total body weight, kg	90 ± 3.5 (55–105)
SOFA score	7 ± 0.5 (4–10)
Albumin, g/L	26.7 ± 1.5 (17.3–42.7)
Bilirubin, µmol/L	15 ± 2.2 (5.5–32.4)
GFR, mL/min	72.3 ± 8.6 (17–125.9)
Loading Dose, mg	200 ± 12.2 (100–250)

Values are presented as median ± standard error (range). GFR, glomerular filtration rate; NA, not applicable; SOFA, sequential organ failure assessment score.

### 2.3 PBPK model evaluation

Model performance was evaluated by comparing simulated plasma concentration-time profiles with published PMB PK data from healthy subjects, patients with ESRD, and ICU populations. For each dataset, a corresponding set of independent virtual trials was generated using dosing regimens identical to those applied in the respective clinical studies (Supplementary Table S1). Inter-individual variability was encoded within the individual parameter distributions, while population-level characteristics, including cohort size and the ranges of age, body weight, and height, were adjusted to align with the demographic profiles of the respective clinical populations.

Sensitivity analysis was conducted using PK-Sim to identify important physiological parameters that affect PMB pharmacokinetics. Model input parameters related to drug distribution and elimination were evaluated for their effects on exposure outcomes, including the area under the plasma concentration–time curve. Sensitivity was calculated using a normalized sensitivity coefficient:

$$S=\frac{\Delta PK/{PK}_{0}}{\Delta P/P_{0}}$$

where S denotes the sensitivity coefficient, PK_0_ represents the initial value of the pharmacokinetic parameter (e.g., AUC), ΔPK is the change in the pharmacokinetic parameter, P_0_ is the initial value of the evaluated model input parameter, and ΔP is the corresponding parameter perturbation. A sensitivity value of +1.0 means that a 10% increase in an input parameter causes a 10% increase in the predicted pharmacokinetic outcome (Willmann et al., 2003). This analysis was used to determine which physiological factors have the strongest influence on PMB exposure.

Noncompartmental analysis (NCA) was performed in PK-Sim to derive pharmacokinetic parameters from the simulated concentration-time profiles. For healthy subjects and patients with ESRD, these parameters were compared with estimates of reported in published pharmacokinetic and population pharmacokinetic studies, including maximum concentration (C_max_), area under the concentration time curve from time 0 (infusion start) to last observed concentration (AUC_0-last_) and CL (clearance). Predicted-to-observed ratios were then calculated by dividing the model-predicted parameter estimates by the corresponding observed values.

For the critically ill patients in addition to comparison of population predicted values, we also compared individual predicted and observed exposure values (AUC_0-last_). Exposure was calculated using NCA in PKanalix 2024R1 (Antony, France). Prediction precision was measured using mean absolute prediction error (MAPE) and root mean squared error (RMSE).

$$MAPE=\frac{1}{n}\times \sum_{i=1}^{n}\frac{\left|{Observed}_{i}-{Predicted}_{i}\right|}{{Observed}_{i}}$$

$$RMSE=\sqrt{\sum_{i=1}^{n}\frac{{\left({Observed}_{i}-{Predicted}_{i}\right)}^{2}}{n}}$$

### 2.4 Clinical data

The PBPK model predictive performance was then evaluated on a prospectively collected dataset on 15 critically ill patients who received PMB therapy and had a range of co-morbidities. This was performed in accordance with the Declaration of Helsinki and national and institutional standards. The study protocol was approved by MEDSI Clinic Independent Ethical Committee, Moscow, Russia (Protocol #29 15 April 2021). Informed consent forms were signed by the patients or their legal representatives.

Clinical and demographic data for the study population is presented in Table 2. PMB was used for the treatment of infections caused by multidrug-resistant Gram-negative bacteria. The loading dose of PMB was selected on the discretion of the treating physician of between 100 and 250 mg in the first 12 h of therapy.

Blood samples were collected throughout the first dosing interval of PMB therapy. 6–8 blood samples (4 mL) were collected in EDTA tubes just before the beginning of PMB infusion, 5 min, 30 min, 1, 2, 4 and 8 h after PMB administration was complete and just before the next PMB infusion (11 h from the completion of the previous infusion). The samples collected were immediately centrifuged for 10 min at 3,470 g and the serum was instantly frozen and kept at −20 °C until the analysis. Serum PMB quantification was performed with the validated direct competitive ELISA method previously developed by our group (Burkin et al., 2021).

### 2.5 Probability of target attainment analysis

The probability of target attainment (PTA) was evaluated using PBPK simulations performed in 1,000 virtual critically ill patients generated in PK-Sim. Simulated plasma concentration-time profiles of polymyxin B were obtained for multiple dosing regimens, including a loading dose followed by maintenance dosing administered every 12 h. The evaluated dosing regimens consisted of 1.5/0.75, 2/1, 2/1.25, 2.5/1.25, 2.5/1.5, 3/1.5 mg/kg for loading and maintenance doses respectively. For each simulated individual, the area under the plasma concentration-time curve over 24 h (AUC_0-24_) as steady state was calculated. Pulmonary interstitial concentrations were predicted using the Rogers and Rowland equations (Rodgers et al., 2005); these predictions were exploratory and were not validated against observed pulmonary concentration data. Pharmacodynamic target attainment was assessed using the AUC_0-24_/MIC index, with a predefined target of *f*AUC_0-24_/MIC >20, consistent with previously reported polymyxin B pharmacodynamic thresholds (Landersdorfer et al., 2018; Tsuji et al., 2019). PTA was defined as the percentage of simulated individuals achieving the PD target at each MIC value. MIC values ranging from 0.1 to 8 mg/L in two-fold increments were evaluated. In addition, the predicted total AUC_0-24_ from venous plasma was assessed against a toxicity threshold of 100 mg h/L (Figure 6) to evaluate the risk of supratherapeutic exposure at each dosing regimen (Yang et al., 2022).

## 3 Results

### 3.1 PBPK in healthy volunteers and patients with ESRD

Existing evidence on the actual biochemical processes related to PMB PK is very sparse, and because of that we introduced some significant assumptions. Available data suggests predominantly non-renal mechanism of PMB clearance (Zamri et al., 2025). Filtration and extensive tubular re-absorption with accumulation of PMB in the kidneys was demonstrated in a pre-clinical study on rats (Manchandani et al., 2016a). PMB urinary recovery is generally low and remains around 1%–5% in heathy volunteers and patients with reduced glomerular filtration (Fang et al., 2024; Liu et al., 2021), as well as critically ill patients (Sandri et al., 2013; Zavascki et al., 2008). PMB demonstrates significant binding with human serum albumin (Poursoleiman et al., 2019; Kaewpaiboon et al.,2022), and the fraction unbound (*fu*) demonstrates significant variability. In healthy volunteers *fu* reached around 5% (Fang et al., 2024), while in critically ill median value was around 40% in most studies (Suro et al., 2022; Sandri et al., 2013; Galvidis et al., 2022), however one study reported a value of ∼15% (Zavascki et al., 2008).

**FIGURE 2:**
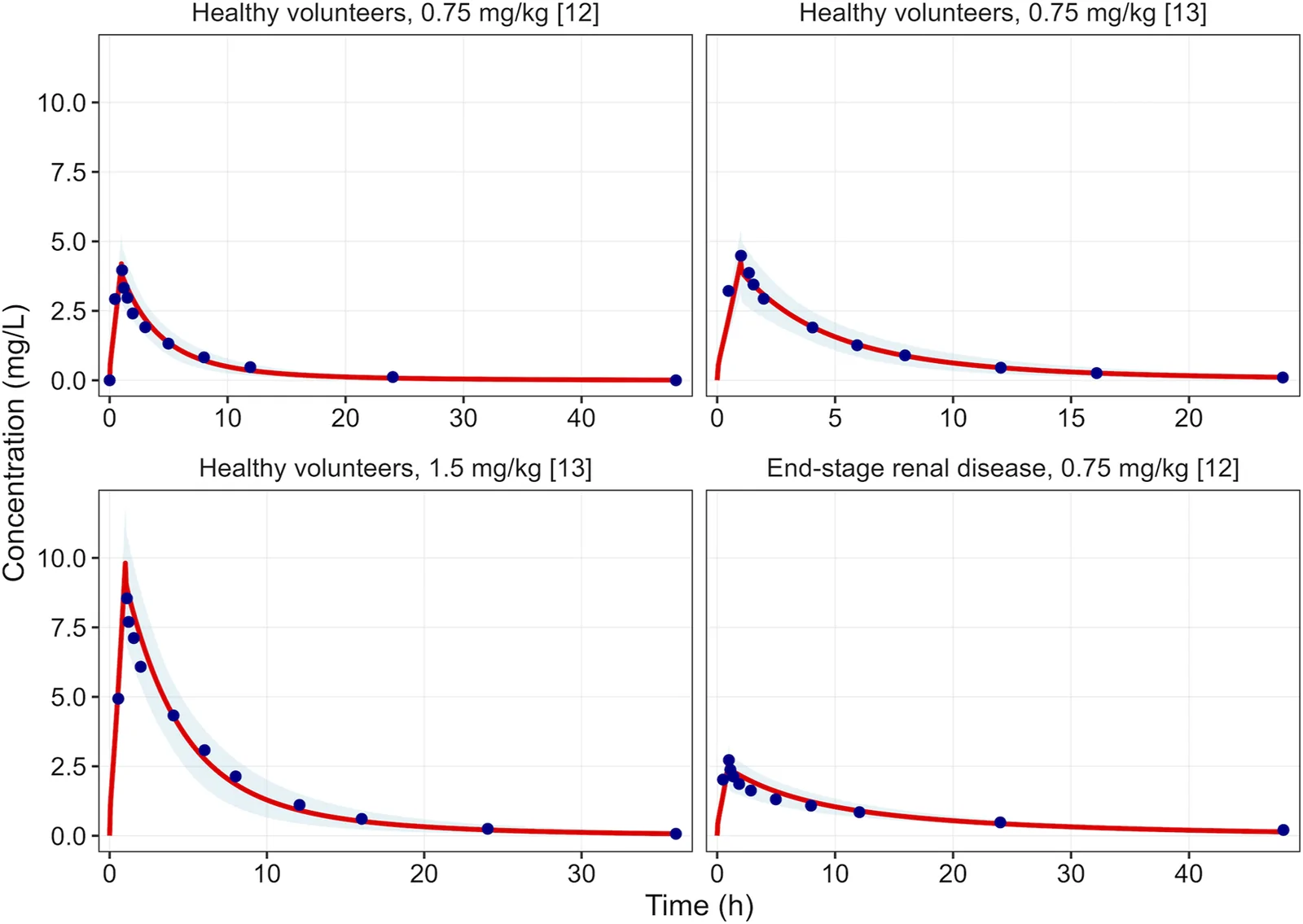
Plasma concentration-time profiles of polymyxin B in healthy populations and end-stage renal disease. Simulated polymyxin B concentration-time profiles were compared with observed clinical data from healthy volunteers receiving 0.75 mg/kg or 1.5 mg/kg, and patients with end-stage renal disease receiving 0.75 mg/kg. Red lines represent the simulated arithmetic mean, shaded areas represent the arithmetic standard deviation range, and blue dots represent observed data.

Simulated plasma concentration-time profiles for PMB in healthy populations are shown in Figure 2 alongside digitised observations from the literature. For the healthy datasets, the model-predicted values of AUC_0-last_, C_max_ and CL were within one standard deviation of the corresponding reported means (i.e., within mean ± SD) from the published pharmacokinetic data. A numerical comparison of simulated and observed pharmacokinetic parameters is provided in Table 3. Across the healthy dataset, predicted-to-observed ratios for AUC_0-last_, C_max_ and CL fell within the ranges 0.89–0.99, 0.90–1.15, 0.97–1.27, respectively.

**TABLE 3:** Non-compartmental analysis for polymyxin B compared observed *versus* predicted.

Parameters	Predicted	Observed	Ratio
Healthy volunteers, 0.75 mg/kg (N = 8, Fang et al. (Fang et al., 2024))			
AUC_0-last_ (h*mg/L)	19.4 ± 4.39	19.2 ± 0.34	0.97
C_max_ (mg/L)	4.19 ± 0.72	3.96 ± 0.65	1.06
CL (L/h)	2.51 ± 0.66	2.59 ± 0.34	0.97
Healthy volunteers, 0.75 mg/kg (N = 13, Liu et al. (Liu et al., 2021))			
AUC_0-last_ (h*mg/L)	21.3 ± 4.73	21.8 ± 1.83	0.89
C_max_ (mg/L)	4.28 ± 0.73	4.66 ± 0.46	0.90
CL (L/h)	2.18 ± 0.58	1.98 ± 0.14	1.27
Healthy volunteers, 1.5 mg/kg (N = 44, Liu et al. (Liu et al., 2021))			
AUC_0-last_ (h*mg/L)	46.71 ± 10.37	47.4 ± 6.39	0.99
C_max_ (mg/L)	9.81 ± 1.37	8.53 ± 1.07	1.15
CL (L/h)	2.03 ± 0.53	1.84 ± 0.28	1.10
ESRD, 0.75 mg/kg (N = 7, Fang et al. (Fang et al., 2024))			
AUC_0-last_ (h*mg/L)	31.4 ± 5.85	31.3 ± 8.04	1.00
C_max_ (mg/L)	2.74 ± 0.56	2.80 ± 0.34	0.98
CL (L/h)	1.47 ± 0.22	1.50 ± 0.39	0.98
Critically ill patients (N = 15, new data)			
AUC_0-last_ (h*mg/L)	26.16 ± 7.15	26.65 ± 8.53	0.98
C_max_ (mg/L)	8.15 ± 1.95	6.77 ± 3.56	1.2
CL (L/h)	8.37 ± 1.7	8.46 ± 2.47	0.99

Data presented as mean ± standard deviation. AUC_0-last_–area under the concentration time curve during the measurement interval, from 0 to 12 h in this case. CL, clearance. C_max_–maximum concentration. ESRD, end-stage renal disease.

Patients with ESRD were characterized by a higher volume of distribution with C_max_ of 2.8 vs. 3.9 g/L in healthy volunteers, as well as reduced CL which was 1.5 L/h vs. 2.9 L/h, respectively (Fang et al., 2024). Urinary recovery of PMB did not differ between the groups. To reproduce the increased volume of distribution observed in dialysis-dependent patients, the interstitial fraction in fat and muscle tissues was increased to 0.3, reflecting tissue oedema in this population (Jaeger and Mehta, 1999), while transporter expression was reduced by 50% to reproduce non-renal clearance reduction, commonly observed in chronic kidney disease (Roberts et al., 2018).

Simulated plasma concentration-time profiles of PMB in patients with ESRD are presented in Figure 2, together with digitized observations from the published data. Although the model predicted a slower distribution phase and a faster clearance phase, all observed concentrations remained within the 2.5^th^-97.5^th^ percentile prediction interval, supporting the validity of the PBPK model. Model-predicted values of AUC_0-last_, C_max_ and CL, were in close agreement with reported values and fell within one standard deviation of the corresponding published means. A quantitative comparison between simulated and observed pharmacokinetic parameters is summarized in Table 3. Predicted-to-observed ratios were 1.00 between simulated and observed pharmacokinetic parameters for AUC_0-last_, 0.98 for C_max_, and 0.98 for CL, indicating good predictive performance of the PBPK model in the ESRD population.

Sensitivity analysis indicated that, in both healthy and ESRD models dose was the most influential determinant of PMB exposure. This was followed by parameters describing transporter-mediated hepatic uptake from plasma into the liver. In contrast, in the ESRD model, the highest sensitivity was observed for blood cell pH, reflecting the population-specific pH adjustment implemented in this population. Other influential parameters in ESRD included the plasma protein scaling factor and unbound fraction, suggesting that alterations in drug ionization and protein binding played a greater role than transporter-related processes. Detailed sensitivity analysis results are provided in the Supplementary Figure S1.

### 3.2 PBPK model in critically ill patients with sepsis

**FIGURE 3:**
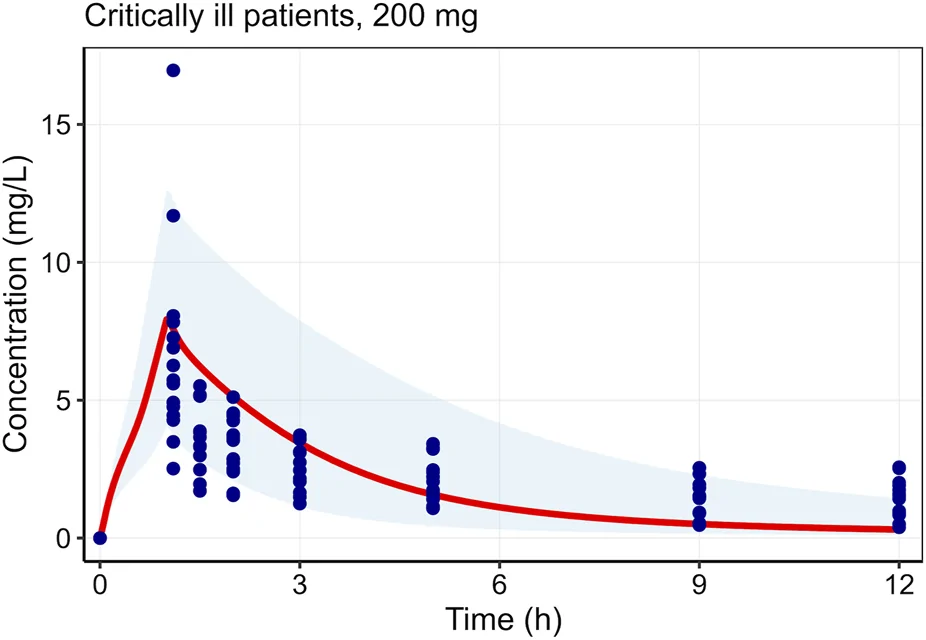
Model prediction of polymyxin B plasma concentration-time profile in critically ill patients. Simulated polymyxin B concentration-time profile was compared with observed clinical data from critically ill patients receiving 200 mg intravenous polymyxin B Red line represents the simulated arithmetic mean, shaded area represents the arithmetic standard deviation range, and blue dots represent observed data.

**FIGURE 4:**
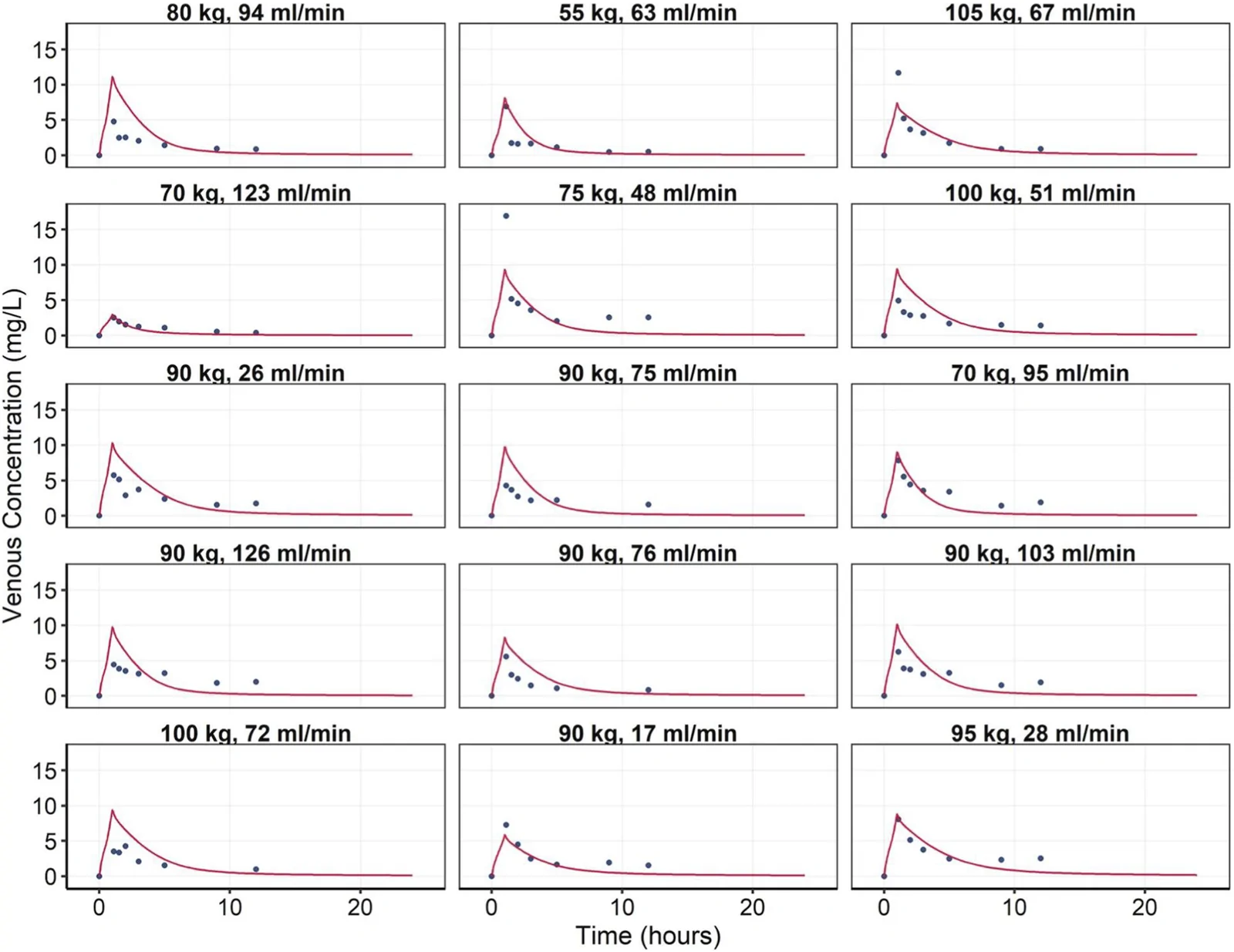
Individual predicted *versus* observed venous plasma concentration-time profiles of polymyxin B in critically ill patients. Each panel represents an individual patient, labelled by body weight (kg) and glomerular filtration rate (GFR, mL/min). Red lines represent model-predicted concentration-time profiles, and blue dots represent observed data.

The population curve predicted for critically ill patients with sepsis using PBPK approach is presented in Figure 3, together with measured concentrations. The model was characterized by a higher predicted C_max_ value which reached 8.15 ± 1.95 mg/L vs. observed C_max_ of 6.77 ± 3.56 mg/L (predicted-to-observed ratio of 1.2), as well as slightly slower distribution phase and steeper terminal elimination slope. Mean exposure value was very similar: AUC_0-last_ (mgh/L) predicted of 26.16 ± 7.15 mg h/L vs. observed 26.65 ± 8.53 mg h/L (predicted-to-observed ratio of 0.98). Individual PK curves are presented in Figure 4.

Sensitivity analyses were performed to evaluate the effects of the unbound fraction, adipose and muscle interstitial fluid fractions and transporter expression levels on the predicted pharmacokinetic parameters. Increasing the unbound fraction from 25% to 45% decreased AUC_0-last_ from 30.28 to 22.47 mg h/L and increased CL from 6.61 to 8.9 L/h. Increasing the interstitial fluid fraction from 0.24 to 0.32 produced a smaller reduction in AUC_0-last_, from 27.80 to 24.53 mg h/L, and a corresponding increase in CL from 7.19 to 8.15 L/h. Similarly, increasing transporter expression from 90% to 110% resulted in only minor changes, with AUC_0-last_ decreasing from 28.48 to 23.85 mg h/L and CL increasing from 7.10 to 8.39 L/h.

**FIGURE 5:**
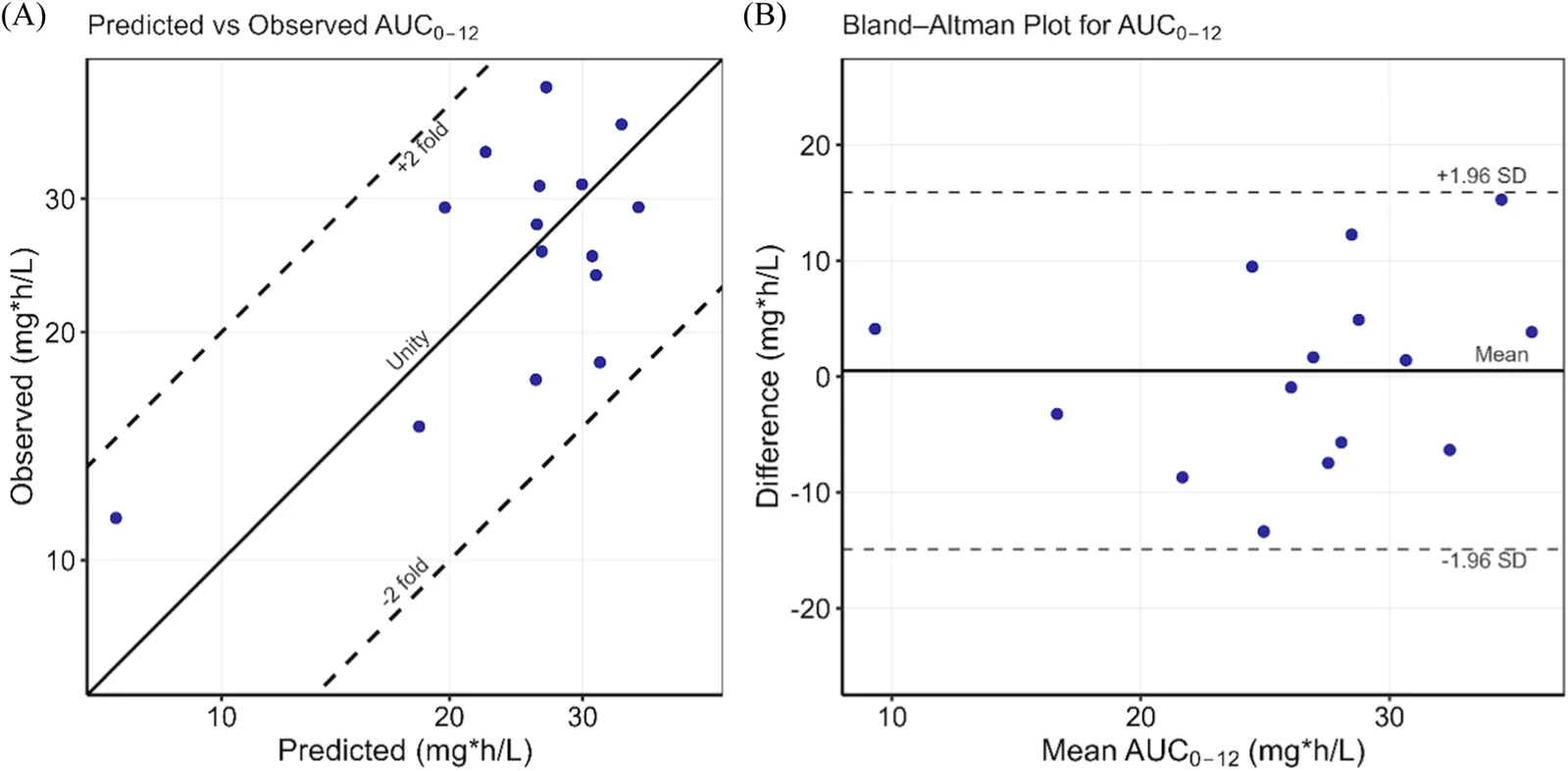
AUC observed in critically ill patients (n = 15) treated with PMB *versus* AUC predicted using the developed PBPK model **(A)** and Blant-Altman plot **(B)** for prediction difference. Left panel: predicted *versus* observed AUC_0-12_ values, where the solid line represents the line of unity and dashed lines indicate the 2-fold error range. Right panel: Bland-Altman plot showing the difference between predicted and observed AUC_0-12_ against their mean, with the solid line representing the mean bias and dashed lines indicating the limits of agreement (±1.96 SD).

In addition, we assessed predictive performance for the individual PBPK models in the critically ill patients with sepsis. The plot of predicted *versus* observed AUC_0-last_ values together with the Bland-Altman plot for prediction difference is presented in Figure 5. The MAPE and RMSE values reached 26.7% and 7.7%, respectively, although the correlation between the observed and predicted values was only moderate (r of 0.47).

**FIGURE 6:**
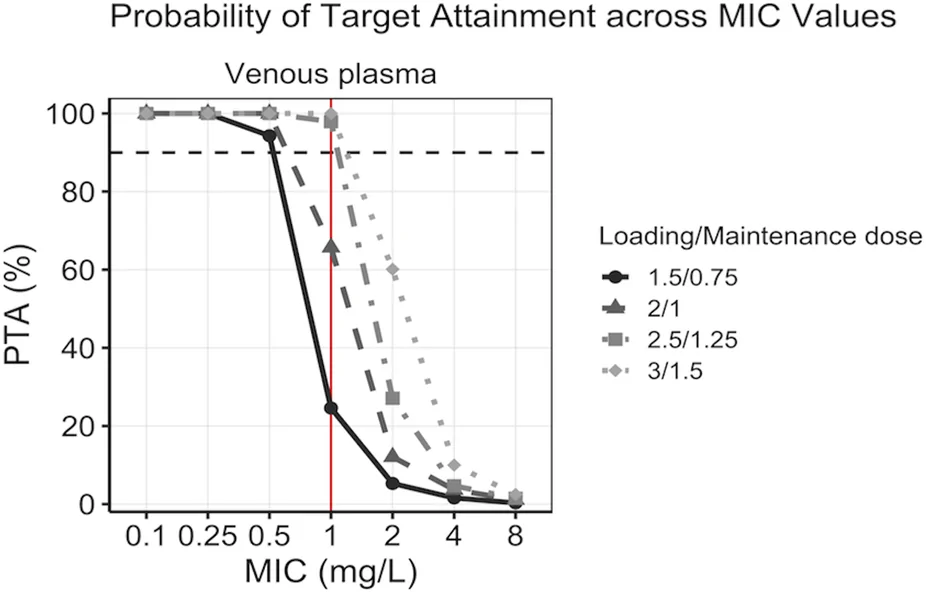
Probability of target attainment (PTA) of polymyxin B across MIC values in venous plasma and pulmonary interstitial fluid. PTA was evaluated for four loading/maintenance dose regimens (1.5/0.75, 2/1, 2.5/1.25, and 3/1.5 mg/kg) in venous plasma. The horizontal dashed line indicates the 90% PTA threshold, and the vertical red line denotes the clinical breakpoint MIC of 1 mg/L.

The PTA for systemic and lung infection was explored for the range of PMB doses (1.5–3 mg/kg loading dose followed by 0.75–1.5 mg/kg maintenance dose) and MICs (0.1–8 mg/L) using the recommended PK/PD threshold for efficacy of *f*AUC_0-24_/MIC >20 (Figure 6). The predicted PMB exposures are presented in Table 4. For systemic infection with MIC ≤0.5 mg/L the PTA >90% was achieved for all dosing regimens, whereas for the MIC 1 mg/L only 2.5/1.25 mg/kg and 3/1.5 mg/kg doses provided sufficient PTA. The target was not reached for any of the dosing regimens with MIC of 2 mg/L and above.

**TABLE 4:** Polymyxin B exposure using PBPK simulations.

Dosing regimen	Plasma *f*AUC0-24		
	P_10_	P_50_	P90
1.5 mg/kg* + 0.75 mg/kg q12 h	10.67	15.20	28.41
2 mg/kg* + 1 mg/kg q12 h	16.21	22.92	44.95
2 mg/kg** + 1.25 mg/kg q12 h	17.04	24.18	49.46
2.5 mg/kg* + 1.25 mg/kg q12 h	22.80	31.30	54.81
2.5 mg/kg** + 1.5 mg/kg q12 h	23.85	34.10	63.35
3 mg/kg* + 1.5 mg/kg q12 h	30.77	43.53	79.78

* 1-h infusion,

** 2-h infusion.

AUC_0-24_ – area under the concentration curve over 24 h; P_10_, P_50_, P_90_–10%, 50% and 90% percentile, respectively; q12 h–every 12 h.

**FIGURE 7:**
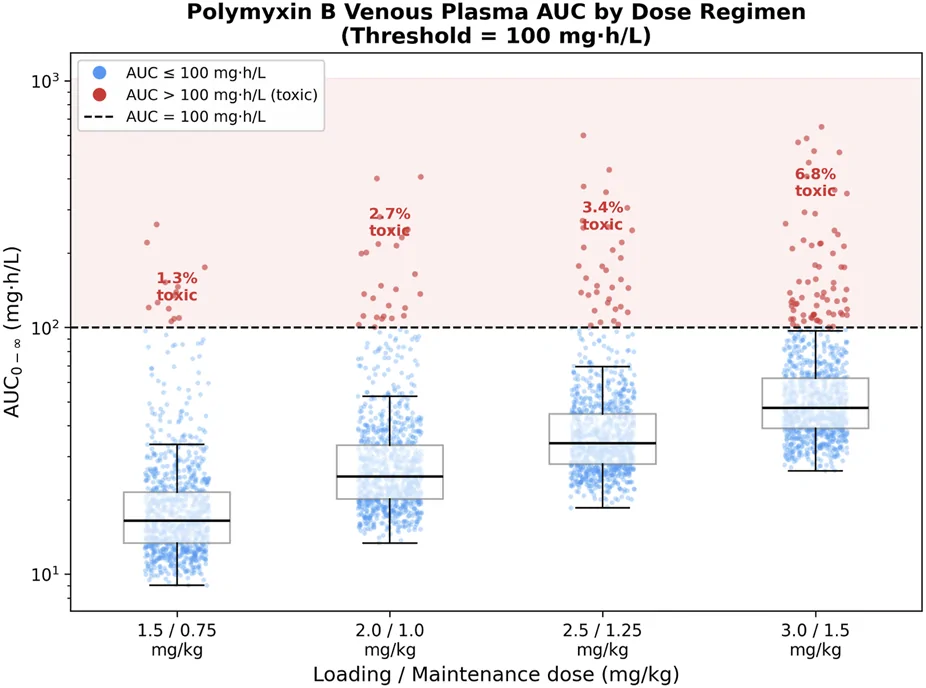
Simulated polymyxin B venous plasma AUC_0-_ ∞ distribution across dose regimens with toxicity assessment. Box-and-whisker plots with individual simulated AUC_0-_ ∞ values are shown for four loading/maintenance dose regimens (1.5/0.75, 2.0/1.0, 2.5/1.25, and 3.0/1.5 mg/kg). Blue dots represent individuals with AUC ≤100 mg h/L, and red dots represent individuals exceeding the toxicity threshold (AUC >100 mg h/L), indicated by the horizontal dashed line. The percentage of virtual patients exceeding the toxicity threshold is annotated for each regimen, with the red shaded region highlighting the toxic exposure range.

For the pulmonary interstitial exposures with same target *f*AUC_0-24_/MIC >20 PT A >90% was achieved for the loading doses of 2–3 mg/kg with MIC of 0.5 mg/L, while for MIC of 1 mg/L the PTA >90% was achieved only with the highest dose (Supplementary Figure S2.). The proportion of simulated patients exceeding the AUC_0-24_ toxicity threshold of 100 mg h/L remained low across the regimens of 1.5/0.75; 2/1; 2.5/1.25; 3/1.5 mg/kg: 1.3%, 2.7%, 3.4%, and 6.8%, respectively (Figure 7).

## 4 Discussion

Infections, caused by resistant microorganisms, remain one of the dominant causes of morbidity and mortality in hospitalized patients (Rudd et al., 2020) with intensive care patients being particularly vulnerable. Once the infection is recognized the management becomes time-critical, and it is vital that the antimicrobial therapy is started early (Seymour et al., 2017) with the right antibiotic (Zasowski et al., 2020; Bassetti et al., 2020), given in the appropriate dose. The risks of antibiotic underexposure pose a significant risk of adverse clinical outcomes (Roberts et al., 2014b), which underscores the significance of pharmacological optimization of antibiotic therapy.

One of the key strategies of antibacterial therapy optimization is usage of a loading dose (Roberts et al., 2014a), which if calculated correctly allows a more rapid achievement of the necessary antibiotic exposure. Typically, the loading dose is calculated by back-extrapolation of the PK model built in patients at steady state. This is also the case for PMB, for which the recommended loading dose of 2–2.5 mg/kg comes from the study performed in a steady conditions (Sandri et al., 2013). Unfortunately, the complex dynamics of critical illness with fluid resuscitation, rapidly changing hemodynamics and wide application of extracorporeal therapy makes “steady state” an elusive condition. With additional constraints imposed by small and highly heterogeneous groups classical population PK analysis becomes extremely challenging.

In these circumstances PBPK may become a valuable complementary method, which would enable prediction of drug behavior in a highly variable system, such as a critical illness. The aim of the current study was to first build a PBPK model calibrated on literature data to predict PMB exposure after a loading dose in critically ill patients with sepsis, then evaluate it on the clinical data and perform the PTA analysis for different doses.

In the current study the PBPK modelling workflow matched the standard bottom-up strategy, from the available physicochemical and animal data to healthy volunteers and patients. To test the model performance in a new setting we specifically did not use available PK data from the critically ill, apart from unbound fraction which is an essential component of PBPK modelling. One of the main difficulties encountered during the PBPK model development was very scarce data from preclinical studies, healthy volunteers and non-critically ill patients. In addition, PMB was treated as a single entity although it is manufactured as a mixture of several components. Although the physicochemical and PK difference are not expected to be clinically-significant (Manchandani et al., 2016b), the manufacturing-related effects cannot be excluded.

Mechanisms of PMB clearance are not entirely understood. The available data supports the following: renal clearance is very low (urinary recovery ∼5%) (Yang et al., 2024; Fang et al., 2024), PMB is found in bile and patients with ESRD have a significant reduction in clearance (Fang et al., 2024). Based on that we assumed a predominantly biliary PMB clearance (also shown for a related substance polymyxin E (Qi et al., 2023), and significant reduction of clearance in ESRD was assumed to be related to decreased transporter expression. With these assumptions the model predicted PMB PK in healthy patients receiving different PMB doses and patients with ESRD (predicted to observed ratios for AUC_0-last_, C_max_ and CL within 1.3).

The model for critically ill accounted for several important physiology alterations: significantly increased unbound fraction (35% vs. 6%), changes of albumin and haematocrit, as well as tissue oedema. The final model allowed good prediction of population mean exposure: predicted AUC_0-last_ value 26.16 mg h/L vs. observed 26.65 mg h/L. Population mean C_max_ values were also close: predicted C_max_ of 8.15 mg/L vs. observed 6.77 mg/L (ratio of 1.2). The MAPE and RMSE for AUC_0-last_ values reached 26.7% and 7.7%, respectively, but model performance in predicting the population variability was modest (r = 0.47). This most likely indicates presence of significant PK-altering factors which are yet to be defined, and in the whole body of PMB PK research there is still significant inconsistency on factors contributing to drug PK variability in critically ill (Zamri et al., 2025).

The developed PBPK model was used to predict PMB exposures with different dosing regimens. To allow the comparison with the previously reported data, which served the basis for PMB loading dose recommendations (Sandri et al., 2013), we included the following regimens: 2 mg/kg loading and 1.25 mg/kg maintenance, 2.5 mg/kg loading and 1.5 mg/kg maintenance. Median *f*AUC_0-24_ values were similar: 24.2 and 34.1 mg h/L in our study vs. 25.6 mg h/L and 33 mg h/L in the comparator study (calculated from the total reported AUC_0-24_ and *fu* of 0.42 in the respective population). The PTA analysis suggest potentially high risk of underexposure with the loading dose below 2.5 mg/kg for the infections caused by microorganism with MIC of 1 mg/L and above, while for the MIC of 2 mg/L and above the evaluated regimens are unlikely to be efficient. The conventional toxic exposure threshold (100 mg*h/L) on the first day of therapy was exceeded in less than 7% patients even with the highest dose tested, although the cumulative effect with prolonged treatment, baselined renal function and actual nephrotoxicity outcomes were not assessed.

One of the important benefits of PBPK analysis is prediction of tissue exposures, and we explored the predicted PMB concentrations in pulmonary interstitium. Model-predicted pulmonary exposures were approximately 2 times lower compared to plasma, and the ratio is similar to the one previously reported two murine pneumonia models (He et al., 2013; Jiao et al., 2023). In the absence of clinical validation, these predictions should therefore be considered exploratory and should not be used to guide dosing. Importantly, current model did not incorporate target-mediated drug disposition resulting from polymyxin B binding to bacterial LPS. This saturable, bacterial-burden-dependent binding may increase total drug retention within infected lung tissue while reducing the freely diffusible fraction, thereby altering the pulmonary concentration-time profile. The quantitative impact of this mechanism in humans remains unknown and needs further investigation.

There are several important limitations of this study. The paucity of preclinical data and no individual-level PK data from the healthy volunteers’ studies limited the refinement of the initial PBPK model. In addition, due to the retrospective design the PBPK model did not include the actual data on hematocrit, markers of tissue edema and unbound fraction, the latter potentially having the most significant impact on PMB PK. Finally, the validation group is relatively small, and the model does not yet allow good prediction of interindividual PK variability, which at present limits its clinical applicability.

To conclude, this study demonstrated a first PBPK model to predict population-level PMB exposure in critically ill and to support the loading dose of at least 2.5 mg/kg. It also highlights feasibility of PBPK modelling in critically ill and the current model could be an important backbone for future PMB PK research.

## Data Availability

The original contributions presented in the study are included in the article/Supplementary material, further inquiries can be directed to the corresponding authors.
