# Correction: Physiologically-based pharmacokinetic model to predict loading dose polymyxin B exposure in critically ill patients with sepsis

**DOI:** 10.3389/fphar.2026.2013189

**Published:** 2026-10-14

**Authors:** Yixuan Cao, Inna Galvidis, Akmal Alimov, Joseph F. Standing, Maksim Burkin, Yury Surovoy

**Affiliations:** 1 University College London, London, United Kingdom; 2 I. Mechnikov Research Institute for Vaccines and Sera, Moscow, Russia; 3 Federal Center for Treatment and Rehabilitation Ministry of Health, Moscow, Russia; 4 Middlesex University, London, United Kingdom; 5 University College London Hospital, London, United Kingdom

**Keywords:** critical illness, loading dose, PBPK, pharmacokinetics, polymyxin B, sepsis

The funder UK Research and Innovation, UKRI885 to JS was erroneously omitted. The corrected Funding statement reads:

“The author(s) declared that financial support was received for this work and/or its publication. This study received funding from UK Research and Innovation, UKRI885, awarded to JS”.

The original version of this article has been updated.

