# Supplementary material for: Physiologically-based pharmacokinetic model to predict loading dose polymyxin B exposure in critically ill patients with sepsis
Source: Front Pharmacol. 2026 Sep 1;17:1827712. doi: 10.3389/fphar.2026.1827712 (PMC13574657; doi:10.3389/fphar.2026.1827712)
Supplement: Supplementary file 1 [file Supplementaryfile1.docx]

SUPPLEMENTARY MATERIALS

**Table S1.** System-dependent parameters for polymyxin B defined in healthy population studies.

| **Reference** | (Yuwei Fang, 2024) | (Xiaofen Liu, 2021) | (Yuwei Fang, 2024) |
| --- | --- | --- | --- |
| **Administration** | IV Infusion (1h) | IV Infusion (1h) | IV Infusion (1h) |
| **Dose (mg/kg)** | 0.75 | a) 0.75 b) 1.5 | 0.75 |
| **Population** | East Asian  (Tanaka, 1996) | East Asian  (Tanaka, 1996) | East Asian  (Tanaka, 1996) |
| **Body Weight (kg)** | 55.3-86.3 | - | 44.1-84.6 |
| **BMI (kg/m^2^)** | 20.9-26.6 | 19-26 | 19.3-26.8 |
| **Age (years)** | 25-43 | 19-45 | 37-59 |
| **% of Female** | 12.5 | - | 42.86 |
| **Number of Individuals** | 8 | a) 13 b) 44 | 7 |

**Sensitivity analysis (Healthy volunteers)**

**Sensitivity analysis (End-stage renal disease)**

**Sensitivity analysis (Critically ill patients)**

**Figure S1. Sensitivity analysis of the PBPK model for polymyxin B across different populations.** Sensitivity coefficients for $AUC_{0\text{–last}}$ are shown for (A) healthy volunteers, (B) patients with end-stage renal disease (ESRD), and (C) critically ill patients. Each point represents the normalised sensitivity coefficient of an individual model input parameter. Parameters with larger absolute sensitivity values exert a greater influence on polymyxin B exposure.

**Figure S2. Probability of target attainment (PTA) of polymyxin B across MIC values in pulmonary interstitial fluid.** PTA was evaluated for four loading/maintenance dose regimens (1.5/0.75, 2/1, 2.5/1.25, and 3/1.5 mg/kg) in pulmonary interstitial fluid. The horizontal dashed line indicates the 90% PTA threshold, and the vertical red line denotes the clinical breakpoint MIC of 1 mg/L.
